# Statistical properties of functional connectivity MRI enrichment analysis in school-age autism research

**DOI:** 10.1016/j.dcn.2025.101534

**Published:** 2025-02-22

**Authors:** Austin S. Ferguson, Tomoyuki Nishino, Jessica B. Girault, Heather C. Hazlett, Robert T. Schultz, Natasha Marrus, Martin Styner, Santiago Torres-Gomez, Guido Gerig, Alan Evans, Stephen R. Dager, Annette M. Estes, Lonnie Zwaigenbaum, Juhi Pandey, Tanya St. John, Joseph Piven, John R. Pruett, Alexandre A. Todorov, for the IBIS Network

**Affiliations:** a Department of Psychiatry; Washington University School of Medicine, 660 S. Euclid Ave, St Louis, MO 63110, USA; b The Carolina Institute for Developmental Disabilities; University of North Carolina at Chapel Hill, 101 Renee Lynn Court, Carrboro, NC 277599-3367, USA; c Children’s Hospital of Philadelphia, University of Pennsylvania, Civic Center Blvd, Philadelphia, PA 19104, USA; d Department of Psychiatry, University of North Carolina at Chapel Hill, Chapel Hill, NC 27514, USA; e McGill Centre for Integrative Neuroscience, McGill University, Montreal, QC H3A 2B4, Canada; f Department of Computer Science and Engineering, Tandon School of Engineering, New York University, Brooklyn, NY 11201, USA; g Department of Radiology, University of Washington, 1410 NE Campus Parkway, Seattle, WA 98195, USA; h Department of Speech and Hearing Sciences, University of Washington, 1701 NE Columbia Rd., Seattle, WA 98195-7920, USA; i Department of Pediatrics, University of Alberta, Edmonton Clinic Health Academy, 11405-87 Avenue, Edmonton, Alberta T6G 1C9, Canada

**Keywords:** Resting state fcMRI, Functional connectivity, Enrichment, Brain Network, Asd, BWAS

## Abstract

Mass univariate testing on functional connectivity MRI (fcMRI) data is limited by difficulties achieving experiment-wide significance. Recent work addressing this problem has used enrichment analysis, which aggregates univariate screening statistics for a set of variables into a single enrichment statistic. There have been promising results using this method to explore fcMRI-behavior associations. However, there has not yet been a rigorous examination of the statistical properties of enrichment analysis when applied to fcMRI data. Establishing power for fcMRI enrichment analysis will be important for future neuropsychiatric and cognitive neuroscience study designs that plan to include this method. Here, we use realistic simulation methods, which mimic the covariance structure of fcMRI data, to examine the false positive rate and statistical power of one technique for enrichment analysis, over-representation analysis. We find it can attain high power even for moderate effects and sample sizes, and it strongly outperforms univariate analysis. The false positive rate associated with permutation testing is robust.

## Highlights

• fcMRI enrichment analysis is a method for conducting brain-wide association studies.
• Addresses problems with significance and power present in mass univariate analysis.
• Can be well-powered for small effects ($R^{2}<0.01$) and low sample sizes (N=50).

## 1 Introduction

Brain-wide association studies (BWAS) are often necessary in developmental neuroscience, when the behaviors and ages being explored lack more developed hypotheses. For functional connectivity MRI (fcMRI) BWAS, a primary limitation arises from multiple testing corrections for the large number of pairs of regions of interest (ROIs), as the threshold for experiment-wide significance scales with the number connections between ROI-pairs tested. A recent analysis called into question the reliability and statistical power of BWAS in smaller samples, and suggested that, using current methodology, sample sizes in the thousands would be required to detect well-powered and reproducible associations between individual ROI-pairs and behaviors (Marek et al., 2022). While there are studies that attain these sample sizes, methods capable establishing reproducible results in more modest and focused samples are also needed.

Enrichment analysis (EA) emerged as a class of methods in high-dimensional genome-wide studies for decreasing dimensionality and increasing statistical power when analyzing the relationship between phenotypic variables and genotype or gene expression (Ackermann and Strimmer, 2009, Das et al., 2020, Tian et al., 2005, Subramanian et al., 2005, Efron and Tibshirani, 2007, Khatri et al., 2012). Enrichment analysis groups individual genes into larger gene sets, characterized by similar functional roles, and aggregates the univariate screening signals for an entire group of genes into a single enrichment statistic.

These same techniques can be applied to fcMRI data and behavioral variables by grouping ROIs into functional network, changing the base unit of inference from the ROI-pair to the network-pair, defined as the set of all ROI-pairs such that the first ROI is in a particular defined functional network and the second ROI is in another defined functional network (or the same, for within network network-pairs).

Several papers applied enrichment analysis to fcMRI data from infants at low- and high-likelihood for developing Autism Spectrum Disorder (ASD), with high-likelihood defined as having a sibling with an ASD diagnosis, through the Infant Brain Imaging Study (IBIS). These studies examined functional connectivity correlates of several autism-related behaviors, including initiation of joint attention (Eggebrecht et al., 2017), walking and gross motor function (Marrus et al., 2018), and subtypes of restricted and repetitive behavior (McKinnon et al., 2019). Enrichment analysis was used to explore ASD heritability by looking at connections between high-likelihood infant fcMRI and multiple measures of their autistic older sibling’s social behavior (Girault et al., 2022). Hawks et al. found a relationship between brain networks associated with error-based learning and ASD-associated behaviors, and summarized many of the primary results from fcMRI enrichment analyses (Hawks et al., 2023).

Outside of the IBIS network, EA has been used to explore motor performance (Wheelock et al., 2018) and attention impairment (Wheelock et al., 2021) correlates in very preterm and term-born children, as well as the interplay between fcMRI correlates of anxiety and attention in the pediatric age group (Perino et al., 2021). It has also been used to establish reproducible site, scanner, and sex effects, in the Adolescent Brain Cognitive Development (ABCD) study (Marek et al., 2019). Separately, an enrichment analysis method directly adapted from the original Gene Set Enrichment Analysis (GSEA) method (Subramanian et al., 2005, Mootha et al., 2003), with a modification to the enrichment score meant to increase statistical power, was used to explore the differences in functional connectivity between participants with (ASD+) and without (ASD-) ASD using a list of functional networks (Cheng et al., 2017). GSEA has also been used with structural MRI data to explore neuroanatomical variations between ASD and other neurodevelopmental disorders (Park et al., 2018). A variation of this method, which uses a generalized additive model (GAM) to more flexibly model brain-behavior interactions, has recently been proposed and tested (Weinstein et al., 2023).

Noble and Scheinost have also introduced the Constrained Network-Based Statistic (cNBS) as a method of functional connectivity inference that acts at the network level, which functions as a method of enrichment analysis which uses the mean of screening statistics as their enrichment statistic (Noble et al., 2020). Using resampling methods on data from the Human Connectome Project (HCP), they have been able to show that it improves power when compared to edge-level and cluster-level methods of inference (Noble et al., 2022). They have also developed a variation of the cNBS which acts on networks derived from edge-level similarity rather than node-level similarity (Rodriguez et al., 2022). However, there has not yet been an examination of the robustness or statistical power of this method for fcMRI data, which is necessary in order to explore whether enrichment analysis actually addresses the difficulties arising from multiple corrections in mass univariate analysis, and thus represents a viable method for conducting connectivity-behavior BWAS. In this paper, we examine the statistical properties of enrichment analysis (i.e., false positive rates and power) using realistic simulations, in a linear model with multiple covariates and nuisance variables. We then compare the power of EA to that of univariate testing. While this method is being developed using data from ASD brain imaging research, we posit that it has wider applicability to research on the associations between functional connectivity and differential behavior expression in other neuropsychiatric disorders.

## 2 Methods

### 2.1 Data used

This study utilized fcMRI and behavioral data from school-aged participants in the multisite Infant Brain Imaging Study (IBIS), a longitudinal study of brain and behavior development following infants at high and low familial likelihood for ASD; that is, participants either have or do not have an older sibling with ASD (Table 1). Exclusion criteria correspond with those in previous IBIS studies (Estes et al., 2015). Informed written assent was obtained for each subject, and consent and parental permission was obtained from at least one parent of all participants, and all study protocols were approved by the University of North Carolina at Chapel Hill’s Institutional Review Board as the lead sIRB site for the IBIS Network.

**Table 1:** Demographic information on subjects. The left column contains the demographics information for all subjects with behavioral data collected, the middle column contains demographics for all subjects with fcMRI data, and the right column contains demographics for all subjects with both behavioral and fcMRI data.

	Behavior Measures Obtained (N = 144)	fcMRI Obtained (N = 121)	Behavior and fcMRI Obtained (N = 105)
High familial likelihood (N, %)	92 (64 %)	78 (64 %)	66 (63 %)
Sex, female (N, %)	56 (39 %)	55 (45 %)	46 (44 %)
Age, years (mean, SD)	10.1 (1.02)	10.0 (1.06)	10.1 (1.01)
ASD Diagnosis* (N, %)	28 (19 %)	21 (17 %)	16 (15 %)
Race, Ethnicity (N, %)	1 (1 %)	1 (1 %)	1 (1 %)
Asian	1 (1 %)	1 (1 %)	0 (0 %)
Black	14 (10 %)	14 (11 %)	11 (10 %)
More than one race	128 (89 %)	105 (87 %)	93 (89 %)
White	6 (4 %)	5 (4 %)	5 (5 %)
Hispanic			

*Clinical best estimate ASD diagnoses were made by trained clinicians; subjects are classified as having ASD if they received a diagnosis at any point during the study.

#### 2.1.1 fcMRI data acquisition and processing

Functional magnetic resonance imaging (fMRI) data were collected from 121 participants at four sites (Children's Hospital of Philadelphia, Washington University in St. Louis, University of North Carolina at Chapel Hill, and University of Washington) at rest while fixating on a crosshair. MRI data was acquired on Siemens (Erlangen Germany) 3 T Prisma and Prisma Fit scanners using a 32-channel head coil. Participants received behavioral training in order to acclimatize them to the scanner environment and minimize participant movement. Additionally, scan duration was intentionally shortened at the cost of signal-to-noise (SNR). Two short-duration T1-weighted images (MP-RAGE; duration: 3:29; 1 ×1 ×1 mm voxels; TE = 2.03 ms; TI = 1000 ms; TR = 2500 ms; flip angle: 8°; acceleration factor: 4) were averaged to increase signal-to-noise while minimizing scan time (Holmes et al., 1998). A short T2-weighted (SPACE; duration: 2:00; 1×1 x 1 mm voxels; TE = 564 ms; TR = 3200 ms; acceleration factor: 4) was also acquired. Four runs of BOLD images (duration: 4:09; Multiband 6; 2.4 ×2.4 ×2.4 mm voxels; TE = 30 ms; TR = 800 ms) for eyes-open resting-state functional connectivity MRI (fcMRI) were acquired. All scans were reviewed by a neuroradiologist. Cross-site functional connectivity differences were not observed.

**Fig. 1:**
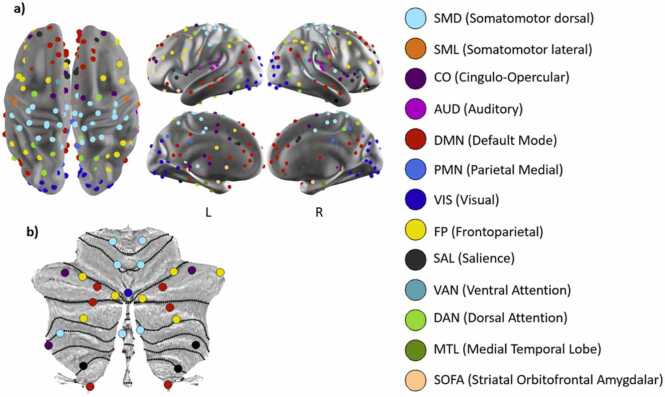
(a) Cortical ROIs, with network assignments for 13 functional networks derived from Seitzman 2020 (Seitzman et al. 2020), are displayed projected onto the surface of the brain (b) Cerebellar ROIs, with network assignments derived from the pre-press version of Seitzman 2020, are displayed onto a cerebellum flat map (Diedrichsen and Zotow, 2015).

Data underwent strict processing procedures analogous to those in previous publications from IBIS. Pre-processing included applying slice timing correction, bias field inhomogeneity correction, mode 1000 image intensity normalization, and rigid body correction for both within-run and cross-run head movement. Subject-level epi distortion correction was estimated and applied using TOPUP, as described in Andersson et al. (Andersson et al., 2003) and as implemented in FSL (Smith et al., 2004). Data were registered to a standard atlas (711–2B version of Talairach space) through a 12-parameter affine transform mapping fMRI -> T2w -> T1w -> atlas image. Post-processing followed methods described in Power 2014 (Power et al., 2014). Data were demeaned and detrended. White matter, CSF, and global signals were used as nuisance regressors. Head movement was quantified and censored at a 0.2 mm frame displacement (FD) equivalent. Data were bandpass filtered at 0.009 Hz < f < 0.08 Hz, and spatial blurring was applied at 6 mm full-width at half maximum (FWHM). Regions of interest (ROI) were selected using Seitzman et. al (Seitzman et al., 2020), although 9 ROIs were excluded due to lack of sufficient SNR. Network assignments for the 230 cortical ROIs were derived from Seitzman 2020 (Fig. 1a) for 13 functional networks, while the non-cortical ROI network assignments came from a pre-press version (Fig. 1b).

It should be noted that the results of enrichment analysis are dependent on the atlas used. Enrichment analysis relies on aggregating results across predefined functional networks, and thus the results are fundamentally tied to the quality of the network atlas. On a computational level, the atlas used determines both the significance level needed to properly control for multiple testing, as well as the power of the method. In this paper, the significance level for an individual enrichment result ($p=10^{-4}$) was selected based on the Bonferroni correction corresponding to experiment-wide significance of p=0.05 when multiply testing 91 network-pairs (the number of pairs between 13 networks). The power to detect a result within a network-pair will depend on the size of the network-pair (the number of ROI-pairs contained within it) as well as the covariance structure of the connectivities within the network-pair. Further discussion of the impact of the functional atlas is in the Discussion.

#### 2.1.2 Behavioral data

This study employed the following behavioral measures: the Autism Diagnostic Observation Schedule, Second Edition (ADOS-II) (Lord et al., 2012), examining both the Social Affect and the Restricted and Repetitive Behaviors calibrated severity scores; the Bruininks-Oseretsky Test of Motor Proficiency, Second Edition (BOT-2) (Bruininks and Bruininks, 2005), employing the Upper Limb subscore; the Multidimensional Anxiety Scale for Children, Second Edition (MASC-2) (March, 2013), analyzing the General Anxiety Disorder index subscore; the matrices non-verbal subtest score from the Differential Abilities Scales, Second Edition (DAS-II) (Elliott, 2007); and the Conners Rating Scale, Third Edition (Conners et al., 2008) inattention subscore. These data were employed as examples of covariate distributions for the purpose of data simulation. A subsequent paper will report scientific results using this data and model (Girault et al. In Preparation).

### 2.2 Enrichment analysis

**Fig. 2:**
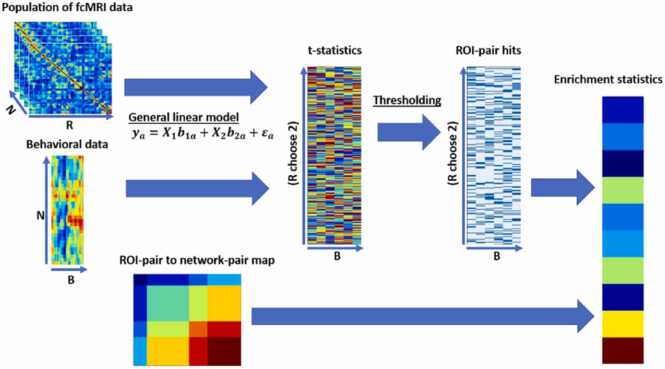
Illustration of fcMRI enrichment over-representation analysis. Mass univariate linear regression is performed on input fcMRI and behavior data. The resultant screening statistics are compared to a predefined threshold to binarize each as a hit or miss. The enrichment statistic is then the number of hits per network-pair. Not shown, these enrichment statistics are then assigned significance through either permutation testing or through comparison to a precalculated enrichment threshold.

An fcMRI enrichment analysis (EA) method first performs mass univariate screening of functional connectivity for each individual ROI-pair and associated behavioral data, followed by an aggregation of the screening results within each network-pair into an enrichment statistic (Fig. 2). A number of enrichment statistics have been proposed. A thorough review is beyond the scope of this paper. See reviews by Das et. al (Das et al., 2020), Ackermann and Strimmer (Ackermann and Strimmer, 2009), and Khatri et. al (Khatri et al., 2012).

Briefly, EA methods fall into two broad categories, depending on the null hypothesis being tested. The "competitive null hypothesis" tests whether enrichment within a network-pair is higher than in the remainder of all ROI-pairs or in another network-pair; whereas the "self-contained null hypothesis" tests if it is higher than would be expected given no association between connectivity and behavior (Ackermann and Strimmer, 2009). Both hypotheses can introduce interpretive difficulties (Tian et al., 2005): the self-contained hypothesis can identify an otherwise statistically identical network-pair as significantly enriched simply due to high association across all ROI-pairs; and (particularly relevant for fcMRI data) the competitive hypothesis can implicate an otherwise unimportant network-pair due to complex covariance structure between the connectivity of ROI-pairs. In practice, the competitive hypothesis is less straight-forward to interpret, and is arguably only relevant if the self-contained null hypothesis has been rejected. In this paper, we focus on the self-contained null hypothesis, leaving the competitive hypothesis for future work.

#### 2.2.1 Mass univariate screening

Here, the relationship between functional connectivity for each ROI-pair and the behavioral data of interest is screened using a simple linear regression model which controls for other behaviors and nuisance variances (here, age, sex, and scan site). More complicated models can be implemented, if warranted (Weinstein et al., 2023).

For each ROI-pair a, we then assume $y_{a}=X_{1}b_{1a}+X_{2}b_{2a}+\varepsilon_{a}$, where $y_{a}$ are the connectivity values for the ROI-pair for N subjects, $X_{1}$ contains the confounding/nuisance variables and intercept term for each subject, and $X_{2}$ contains the behavioral variables of interest for each subject; here, the six behavioral variables described in Section 2.1.2.

Connectivity is thus modeled as a function of behavior. Previous versions of enrichment analysis for fcMRI studies modeled behavior as a function of connectivity. This switch is made for practical reasons. The distribution of connectivity given behavior is far more well-behaved than the converse. Behavioral variable distributions are often zero-inflated, long tailed, or otherwise ill-distributed. Modeling a single behavioral variable is feasible, with an appropriate link function, but modeling several jointly becomes arduous, if not intractable. On the other hand, connectivity is relatively normally distributed, and the permutation distributions of *t*- statistics from linear models of connectivity regressed on behavior closely follow those expected under the null. There should be no difference between the different two approaches, provided the joint likelihood could be correctly specified. Since this is not the case, we can expect some differences between the two.

The screening statistics in this enrichment analysis are the individual *t*-statistics from the above linear model (here, 6 statistics per ROI-pair, one per covariate). Some previous enrichment methods transform the screening statistics, such as with a moderated *t*-statistic (Wu and Gordon, 2012) or z-transformed statistics (Kim and David, 2005). A full discussion of the effect of these transformations appears in Ackermann and Strimmer (2009). In the present study, these transformations were unnecessary. Here, we use one-sided screening; enrichment for a covariate is thus here characterized as a larger number of positive associations (after controlling for the effect of other covariates) than expected under the null hypothesis.

#### 2.2.2 Over-representation analysis (ORA)

Several enrichment statistics have been proposed. In over-representation analysis (ORA), the enrichment statistic is simply the number of screening statistics in a network-pair that lie above a predefined threshold, or within a particular quantile for all ROI-pairs. Here, α indicates quantile of the screening statistic distribution set as the ORA threshold; i.e. α=0.95 would indicate that the top 5 % of the screening statistics are counted as hits, while α=0.50 would count any screening statistic above the median as a hit; given that the *t*-statistics here follow the expected null *t*-distribution, any positive screening statistic would be considered a hit.

Rigorously defined, if $N_{i}$ is defined as the set of ROIs (a) assigned to the *i*-th functional network, then the *i,j*-th network-pair is defined as $N_{\mathrm{ij}}=\{\left(a,b\right)\;:a\in \;N_{i}\;,b\in N_{j}\}$, or the set of all unordered pairs of ROIs such that the first ROI belongs to the *i*-th functional network and the second ROI belongs to the *j*-th functional network.

There are numerous examples where ORA performs well in genomics (Nurnberger et al., 2014, Wang et al., 2009, Menashe et al., 2010), as well as in some of the fcMRI studies cited above in Section 1. Common objections to ORA are the arbitrariness of threshold selection, and potential information loss due to reducing the screening statistics to binary hits/misses (Das et al., 2020). We will explore the impact of threshold choice on false positive rates and statistical power. Another criticism is that the reported false positive rate can be inflated when not accounting for the correlation between screening statistics (Mathur et al., 2018, Tarca et al., 2013). This criticism is primarily relevant to methods which determine significance without permutation testing, e.g. through a hypergeometric or binomial test (Goeman and Bühlmann, 2007). Here, we verify that the reported false positive rate is robust when using permutation testing.

In contrast, functional scoring (FCS) uses (semi) continuous measures of enrichment, such as the *max-mean* statistic (Efron and Tibshirani, 2007), the Wilcoxon rank sum (Barry et al., 2005), or a Kolmogorov-Smirnov statistic (Subramanian et al., 2005), amongst others. A good summary of FCS methods is provided by Khatri et al. (Khatri et al., 2012). Newton et al. concluded that ORA should perform better for a set (here, a network-pair) containing relatively few elements (here, ROI-pairs) of large effect, whereas FCS should perform better in a set with a larger number of constituent elements with smaller individual effect (Newton et al., 2007). In this paper, we focus on ORA. An examination of FCS will be conducted in the future.

### 2.3 Distribution of enrichment statistics under the null

There is no simple form for the distribution of the enrichment statistic within a network-pair due to the complex covariance structure of fcMRI data and the large dimensionality of most network pairs. Significance testing for EA thus typically proceeds with permutation tests. Permuting complete phenotypes breaks the connectivity-phenotype association while maintaining the covariance structure for both the connectivity and the phenotype variables (Backes et al., 2014). Here, our focus is on the effect of behavior *after* accounting for nuisance variables. We then use permutation tests as described in Solari et. al (Solari et al., 2014), which improves on the Freedman-Lane procedure (Freedman and Lane, 1983). Permutation testing relaxes many assumptions that parametric methods make about the dependence structure and distributions of the errors, making it more applicable to data with complex covariances structure, such as neuroimaging data (Winkler et al., 2014). Winkler et al. compared a number of permutation testing methods, and suggest Freedman-Lane for neuroimaging data. Solari et al. present the method in the context of rotation tests, a variant of permutation testing originally described in Langsrud (Langsrud, 2005) and presented as a method for significance assessment for enrichment analysis in ROAST (Wu et al., 2010). However, we found no difference between permutation and rotation tests in this work and utilized permutation matrices for ease of implementation.

In a comparison of various enrichment analysis methods, it was found that the p-value returned through permutation testing did not always correspond with the observed false positive rate (Mathur et al., 2018), particularly in methods which fail to account for the correlation between screening statistics. While this enrichment analysis does account for such correlations, the robustness of permutation testing as a method for significance assessment for enrichment analysis on fcMRI data needs to be verified.

In order to verify the robustness of permutation testing, as well as to expedite the later power computations, an initial round of simulations was run in order to compute enrichment statistic significance cutoffs. These cutoffs will determine whether an enrichment statistic is statistically significant without needing to run a permutation test, the most computationally expensive part of the enrichment analysis. This is not a step that would need to be taken for a researcher carrying out enrichment analysis. To clarify possible confusion, these enrichment statistic significance cutoffs are different from the screening statistic threshold (α), which is used in computing the ORA enrichment statistic.

In order to compute these enrichment statistic significance cutoffs, 1000 random pairings of 105 of the 121 available fcMRI matrices and 105 of the 144 behavioral vectors were generated; there were 105 subjects with both behavioral and fcMRI data, making this a natural sample size to simulate. There are $∼10^{222}$ such random pairings. This method of data generation breaks any correlation between connectivity and both behavior variables and nuisance variable (age, sex, site). The permutation distributions of the ORA statistics were estimated in each of the 1000 datasets, using 1 million permutations each. The permutation distributions of the ORA statistics were estimated in each of the 1000 datasets, using 1 million permutations each.

The resultant CDFs were used to obtain a distribution of enrichment statistic significance cutoffs for a target statistical significance level of $p\leq 10^{-4}$ (roughly the Bonferroni correction for experiment-wide significance of 0.05 over 91 network-pairs). The 75th quantile of this distribution was then selected as the significance cutoff for the rest of the project. These cutoffs were then validated by estimating the false positive rate, first on an additional 500,000 random pairings of 105 fcMRI and behaviors. We then validated them on 500,000 simulated samples with three behavioral variables, drawn from Gaussian, uniform, and a thresholded Gaussian respectively.

### 2.4 Power computations

The aim was to simulate functional connectivity data with a covariance structure similar to that observed in our data, while adding an embedded connectivity/behavior effect. For a given network-pair, the method employed generates randomized connectivity data for a chosen sample size (N) with covariance derived from the observed data, then generates a randomized behavior variable, and then adds a deviation to the connectivity data such that there is an association between the generated connectivity and behavior data. The strength of this association is able to be varied, characterized by the mean and standard deviation, (μ,σ), of the regression coefficients, as described below.

Data were simulated for a single network-pair at a time; because this enrichment analysis uses the self-contained null hypothesis, computation of the enrichment statistic and significance is fully independent for each network-pair, and so, these power computations were carried out independently for each network-pair. We did not assume that the strength of the relationship between connectivity and behavior was the same for all ROI-pairs within a network-pair (an unrealistic assumption). The regression coefficients relating behavior to connectivity for each ROI-pair were then assumed to be independently normal with mean µ and standard deviation σ. Roughly stated, µ indicates the average strength of the association between behavior and connectivity for ROI-pairs in a network-pair; and σ, its variation across ROI-pairs. We thus entertain scenarios such as a mean effect of zero, while still having some associated ROI-pairs with a potentially high effect. In Results, we outline the implications of this model in terms of behavior variance explained. For a network-pair with *R* constituent ROI-pairs and a sample size of N:

1. Estimate the R×R connectivity covariance matrix Ω from observed fcMRI data using the Ledoit-Wolf algorithm (Ledoit and Wolf, 2004) and its Cholesky decomposition C
2. Generate R regression coefficients (behavior/connectivity effects) for the ROI-pairs, B, from a normal distribution of mean μ and standard deviation σ
3. Generate the N×1 behavior scores, X, drawn from a unit normal
4. Simulate the N×R functional connectivity data as $f=\mathrm{XB}+ZC^{T}$, with the N×R elements of Z i.i.d. N(0, 1)

The Ledoit-Wolf algorithm was used since N≪R for most network pairs (Ledoit and Wolf, 2004). We let N range from 25 to 200 and considered mean effect size from μ=0 to μ=1.5 and standard deviation from σ=0 to σ=1.5. 5000 sets of data are generated for each model and sample size. Power was estimated for 10 different screening statistic thresholds between α=0.50 and α=0.95. We note that, here, we are estimating power for a single covariate.

**Fig. 3:**
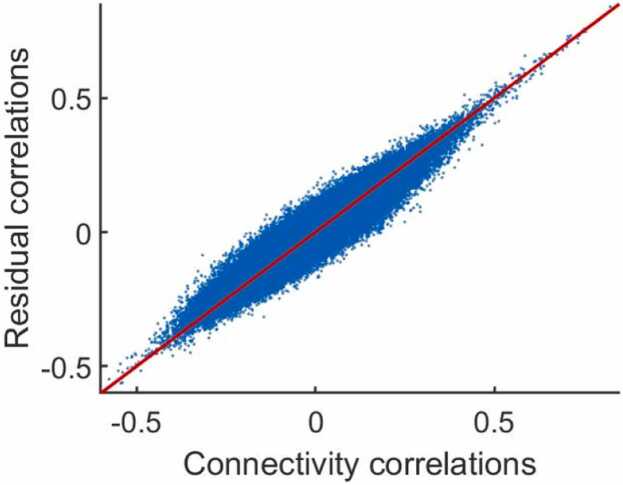
Covariance structure is not drastically affected by using raw connectivity values as opposed to controlling for behaviors first. For 1000 random ROI-pairs plotting correlations between observed connectivity against correlations between residuals when regressing connectivity on behavior in actual data.

This simulation method could over-estimate the residual correlations, due to behavior-connectivity effects in the original data. However, the implied residual correlations were not meaningfully different from those computed using the residuals from a regression of connectivity on behavior in the actual dataset (Fig. 3). This is for one dataset only, but provides some confidence in the realism of this simulation method.

### 2.5 Implementation

Enrichment analysis was implemented in Python and Fortran. Simulations were run on UNC's Longleaf computing cluster. Runtime for a single iteration of enrichment analysis on a single network-pair using one core depends on sample size and number of constituent ROI-pairs. For a network-pair of size 200, a single run took 16 seconds for a sample size of 100, and one minute for a sample size of 500. For the largest network-pair, of size 3500, a single run took 14 min for a sample size of 100 and an hour for a sample size of 500. The full power analysis, parallelized across 700 cores, required around a day of runtime.

Applying the method to a single fcMRI/behavior dataset can be accomplished on a single machine within around an hour. Carrying out a power analysis is far more computationally expensive, likely requiring access to cluster computing resources, although it is greatly expedited by estimating significance thresholds as done here.

## 3 Results

### 3.1 ORA is unsuitable for smaller Network-Pairs

**Fig. 4:**
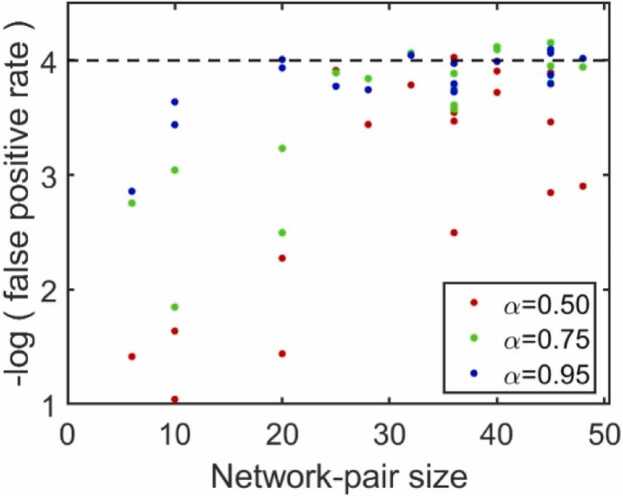
Statistical significance can be unattainable for smaller network-pairs. The negative logarithm of false positive rates (determined using previously computed enrichment statistic thresholds) for network-pairs with fewer than 50 constituent ROI-pairs are plotted against the size of the network-pair, colored by the screening statistic threshold used. The target false positive rate ($p=10^{-4}$) is indicated by the black dashed line.

As an extreme example, the MTL-MTL network-pair has only 6 constituent ROI-pairs. The probability that all 6 would be hits, strictly by chance, is ∼0.04. An experiment-wide significance level of $10^{-4}$ (broadly corresponding to Bonferroni correction for experiment-wide significance of 0.05 when testing 91 network-pairs) simply cannot be attained (Fig. 4). We excluded 19 smaller (fewer than 50 constituent ROI-pairs) network-pairs (out of 91 total) from the power computations, where ORA was unable to attain appropriate significance for at least one screening threshold level. Alternative enrichment methods, such as FCS, may be better suited for smaller network-pairs.

### 3.2 Significance cutoffs

**Fig. 5:**
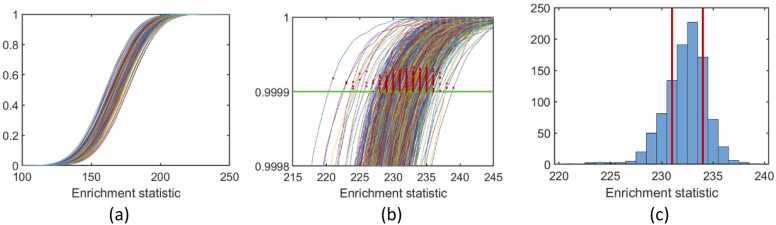
An illustration of how enrichment statistic cutoffs were determined. Panel 1 shows the enrichment statistic CDFs for 1000 generated datasets (N=105), comprised of a million permutations each, for the DMN-SML network-pair (size S=336) and BOT-2 upper limb subscore, and screening statistic threshold α=0.5 (i.e., screening statistics in the top 50 % of the distribution count as hits, corresponding to a positive screening statistic). Panel (b) shows the selection of the first enrichment statistic associated with p≤0.0001 for each CDF. Panel (c) shows the distribution of these values, with the official enrichment statistic cutoff selected as the 75th quantile, with the 25th quantile selected as an alternate cutoff to explore the effect of quantile choice. A range of cutoffs between 230 and 235 for a network-pair of this size is reasonable. (Key: DMN = Default Mode, SML = Somatomotor Lateral).

We estimated the ORA statistic cutoff associated with the 0.0001 level of significance for each network-pair, behavioral variable, and screening statistic threshold (Fig. 5, for one example). This was done for 1000 randomly paired data sets (detailed in Sections 2.3), and 1 million permutations per dataset.

We expect some variation in the cutoff estimates, especially in the extreme tail ends (Fig. 5). However, this variation was not dramatic. For example, in Fig. 5c, the 25th and 75th quantiles differ by only 3 ROI-pairs (out of 336 total ROI-pairs in the DMN-SML network-pair). The 75th quantile of the estimated cutoffs, over the 1000 datasets, was used as the ORA statistic cutoffs for the rest of this project. Results were not drastically affected by the use of the 75th percentile compared to a more liberal, say, 25th percentile.

To verify that these cutoffs were applicable to other sample sizes, we reran the previous simulations for N=25 and N=50 for 5 sample network-pairs and compared the resultant cutoffs to those for N=105, with 100,000 randomly paired datasets each. This resulted in less than 2 % relative change in the cutoff estimates.

These cutoffs appear broadly unaffected by differing behavior variable distributions. Among the six observed behavioral covariates, cutoffs varied by an average of 0.9 % relative change, with a maximum of 3 % relative change. Including the three simulated covariates, cutoffs varied by an average of 1.3 % relative change, with a maximum of 5 % relative change.

These cutoffs were used in a validation run, using 500,000 randomly paired datasets for each network-pair, behavioral variable, and screening statistic threshold. Using the conservative 75th percentile cutoffs, we obtain a mean false positive rate of 0.0009(±0.0003), very near the target significance of $p=10^{-4}$. With the more liberal 25th percentile cutoffs, we obtain a mean false positive rate of0.0018(±0.0005). Permutation testing appears to be a robust method significance assessment of this enrichment analysis and does not seem to inflate the reported false positive rate.

### 3.3 ORA enrichment analysis statistical power

The connectivity and behavior data used for these power analyses are simulated so as to have a similar connectivity covariance structure as observed data, and with an embedded connectivity/behavior effect, with the regression coefficients across all ROI-pairs within a network-pair being normally distributed, with mean μ and standard deviation σ.

**Fig. 6:**
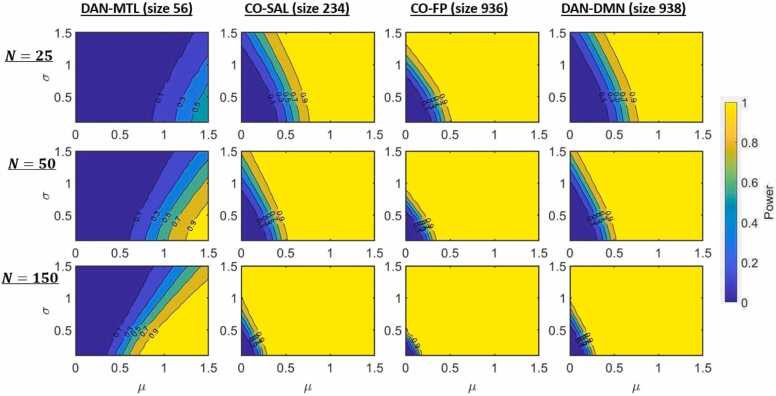
Simulated power to detect an embedded connectivity/behavior effect (characterized by mean (μ) and standard deviation (σ) of the effect across ROI-pairs) for various sample sizes and network-pairs. The top row has sample size N=25, the middle row N=50, and the bottom row N=150. The columns, from left to right, use the DAN-MTL network-pair (of size R=56 ROI-pairs), the CO-SAL network-pair (R=234), the CO-FP network-pair (R=936), and the DAN-DMN network-pair (R=938). All plots here use a screening statistic threshold of α=0.90. (Key: DMN = Default Mode, CO = Cingulo Opercular, DAN = Dorsal Attention, MTL = Medial Temporal Lobe, SAL = salience, FP = Fronto-Parietal).

As expected, power increases with μ and sample size, but the rate of increase will depend on network-pair characteristics, screening statistic thresholds, and σ (Fig. 6). For the same (µ, σ), power generally, but not always, increases with the number of ROI-pairs in a network-pair, and the transition region (in terms of power) becomes sharper. However, this is not universally true; two network-pairs with nearly identical sizes can have largely different power, due to differing covariance structures. The precise relationship between power and network-pair properties (size and covariance structure) will be explored in future work.

**Fig. 7:**
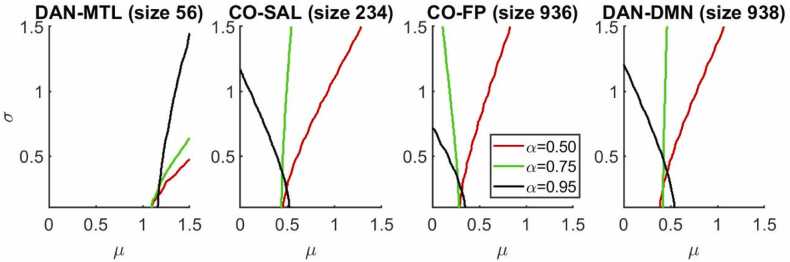
As screening statistic threshold increases, power increases for most embedded effects, though power may decrease for effects with sufficiently small deviation across ROI-pairs (σ). The contour corresponding to 80 % power is shown for four network-pairs and a sample size of N=50, for three screening statistic thresholds, from α=0.50 (any positive association is counted as a hit) to α=0.95 (top 5 % of associations are counted). That is, a connectivity/behavior effect characterized by (µ, σ) to the right of a contour will be detected by ORA enrichment analysis with corresponding screening statistic threshold with a power greater than 80 %. (Key: DMN = Default Mode, CO = Cingulo Opercular, DAN = Dorsal Attention, MTL = Medial Temporal Lobe, SAL = salience, FP = Fronto-Parietal).

In general, as we increase the screening statistic threshold for computing ORA enrichment statistic, the power in detecting a particular connectivity/behavior effect increases (Fig. 7). This is most noticeable for effects characterized by high variation across ROI-pairs (high σ). However, for a signal characterized by low σ (comparable signal across ROI-pairs), power can be higher for a lower screening statistic threshold. For most situations, a higher threshold (α=0.95) is a safer bet, although if the effect is expected to be more homogenous across ROI-pairs, a lower threshold may work better. Additionally, it should be noted that as the screening statistic threshold approaches 1, power will sharply drop off to 0, as no screening statistics will be high enough to register as hits.

**Fig. 8:**
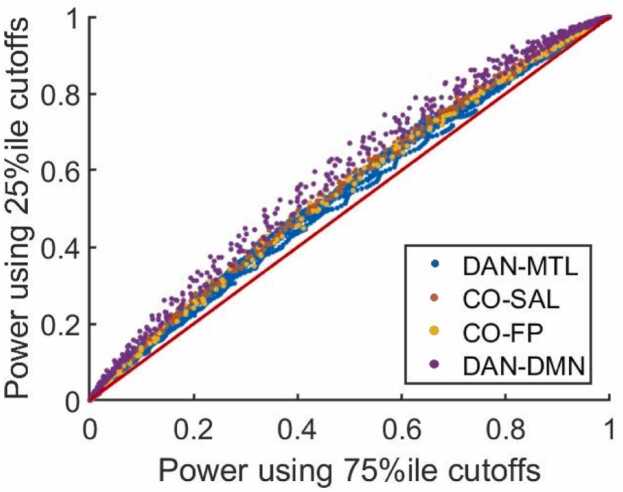
Power is not drastically affected by deriving the ORA enrichment statistic cutoffs for statistical significance from the 25th vs the 75th percentile of the previously found distributions of significance cutoffs. For four network pairs, a range of embedded effects, and sample size N = 50, the power of ORA enrichment analysis using the cutoffs derived from the 75th percentile of the previously found distributions are plotted against the power in detecting the same effect using the cutoffs derived using the 25th percentile. The line of parity is also shown in red. (Key: DMN = Default Mode, CO = Cingulo Opercular, DAN = Dorsal Attention, MTL = Medial Temporal Lobe, SAL = salience, FP = Fronto-Parietal).

The ORA enrichment statistic cutoff for statistical significance was previously selected as the 75th quantile of the distribution of computed significance cutoffs. We found that power was not drastically affected by selecting the 25th quantile. Power increases slightly by using the more liberal 25th percentile (Fig. 8), but not to a degree that qualitatively alters any of our results. For effects with power in the transition region (defined here as above 0.05 and below 0.95), across all network-pairs and sample sizes, there was a mean increase of power by 0.065, with a maximum increase of 0.3, from 0.47 to 0.77.

### 3.4 Power simulation model implications: (µ, σ) to $R^{2}$

The method used for generating simulated connectivity data characterizes the embedded connectivity/behavior effect through (µ, σ), the mean and standard deviation respectively of the regression coefficients across ROI-pairs within a network-pair. We explore their connection to the expected coefficient of determination ($R^{2}$).

**Fig. 9:**
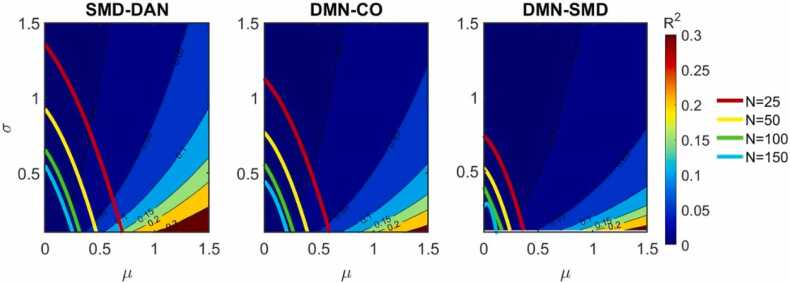
Enrichment analysis attains high power even for effects with low coefficient of determination. For three network-pairs (with network-pair size R=742, 1742, and 3551 respectively), expected $R^{2}$ is displayed as a function of µ and σ. Additionally, overlaid on top of this, the contour corresponding with 80 % power is displayed for various sample sizes. That is, a connectivity/behavior effect characterized by (µ, σ) to the right of the yellow contour will have a corresponding ORA enrichment power greater than 80 % for a sample size of N=50, but an effect characterized by a (µ, σ) to the left of the red contour will have an ORA enrichment power less than 80 % for a sample size of N=25. (Key: DMN = Default Mode, SMD = Somatomotor Dorsal, CO = Cingulo Opercular, DAN = Dorsal Attention).

We see that, within the ranges of µ and σ that we have been exploring, the expected $R^{2}$ reflect the moderate values that are often observed in neuroimaging association studies. Combining these results with the power estimates (Fig. 9), we can see that ORA enrichment analysis can be well-powered (over 80 % power) for very mild effects, where connectivity across the ROI-pairs in a network-pair accounts for a small percentage of the behavior variance ($R^{2}<0.01$).

### 3.5 Comparison to univariate power

There is no simple way to compare power for ORA (which performs 91 tests here) to power for mass univariate analysis (around 42 K tests). Here, we try to give a sense of what (µ, σ) mean in terms of univariate power, using a very liberal significance level ($p=10^{-3}$) to be favorable to univariate analysis, while acknowledging that most such findings in a mass univariate screening would be false positives.

**Fig. 10:**
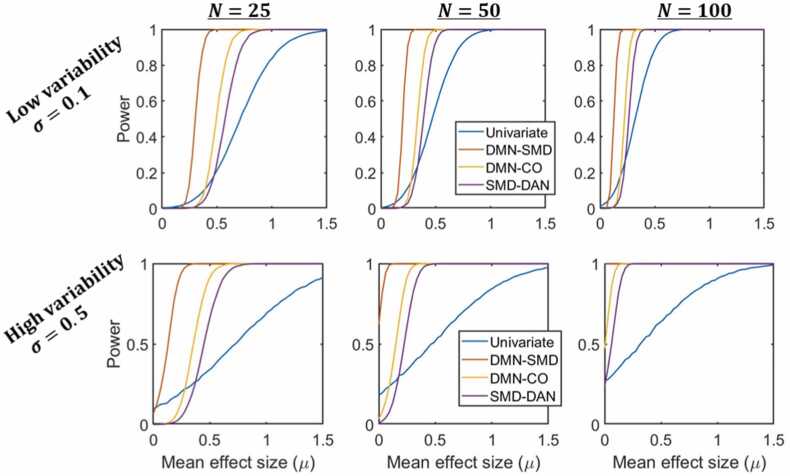
ORA enrichment analysis is well-powered for milder effects than univariate analysis. Comparisons between the power of univariate analysis (for p=0.001) on a single ROI-pair in detecting an embedded signal in simulated data and the power of enrichment analysis (for α=0.95) in detecting an equivalent signal in three different network-pairs of various sizes (S=3551, 1742, and 742 for DMN-SMD, DMN-CO, and SMD-DAN respectively), for three sample sizes (in the left column N=25, in the middle N=50, and in the right N=100). The statistical power is plotted against the mean effect size (μ), for univariate analysis and for enrichment analysis on each network-pair, for two effect size standard deviations, σ=0.1 on the top row and σ=0.5 on the bottom. (Key: DMN = Default Mode, SMD = Somatomotor Dorsal, CO = Cingulo Opercular, DAN = Dorsal Attention).

Fig. 10 shows a comparison between the power of univariate analysis and enrichment analysis (for screening statistic threshold α=0.95) for several network-pairs of various sizes, across all values of µ and σ used. While univariate analysis occasionally outperforms enrichment analysis when both have low power, enrichment analysis quickly outperforms univariate analysis, reaching a power of 1 while univariate analysis still has a power below 0.25. Even using an unrealistically liberal experiment-wide significance threshold for univariate analysis, enrichment analysis is still well-powered for effect sizes and sample sizes where univariate analysis is woefully underpowered. This is most apparent for effects with larger σ values, that is, higher effect variability across ROI-pairs.

This does not hold for all network-pairs. As mentioned in Section 3.1, ORA is not suitable for smaller network-pairs. There are some instances where univariate analysis outperforms enrichment analysis and attains high power for a milder effect than enrichment analysis. This typically occurs in smaller network-pairs (those with fewer than 100 ROI-pairs) with a lower variation of effect size across ROI-pairs (σ<0.5). For N=100, univariate analysis reaches 80 % power for a lower µ than enrichment analysis for 22 % of network-pairs for a low variation effect (σ=0.1) and only 1 % of network-pairs for a higher variation effect (σ=0.5)

## 4 Discussion

Enrichment analysis is a promising method for performing fcMRI BWAS that addresses the statistical problems that come with mass univariate analysis, such as low power and reproducibility (Marek et al., 2022). While these methods have been used and explored extensively in genomics research (Ackermann and Strimmer, 2009, Mathur et al., 2018), there is little work (Weinstein et al., 2023) exploring their statistical properties when applied to fcMRI data.

The particular enrichment analysis method employed in this work improves on that used in previous applications to fcMRI data (Eggebrecht et al., 2017, Girault et al., 2022). By modeling connectivity as a function of behavior, rather than vice versa, in the screening stage, we were able to simultaneously test multiple covariates of interest while controlling for nuisance or confounding variables, despite ill-behaved covariate distributions.

We found that permutation testing on an ORA enrichment statistic is robust. The present method is able to attain high power for detecting moderate brain/behavior effects for low sample sizes, and outperforms univariate analysis, even with an unrealistically liberal significance threshold for univariate analysis. However, we also found that ORA was often unsuitable for network-pairs with fewer than 50 constituent ROI-pairs.

It is worth noting that enrichment analysis is designed specifically for brain-wide studies, intended primarily for discovery neuroscience. At the heart of enrichment analysis is a trade-off between spatial specificity and power, aggregating results over functional networks in order to increase power at the cost of any resolution finer than the network level. Enrichment analysis would thus not be well suited for a study seeking to find associations on a more localized scale, which would require a more targeted hypothesis and analysis method.

The methods provided here can be used both to analyze existing fcMRI data, as well as to perform power analyses to advise on future study design. We suggest selecting the significance level using the Bonferroni correction for the number of behaviors and network-pairs explored. To perform prospective power analyses to advise on sample size, a set of rs-fcMRI data from the relevant population is needed, as well as determining a network-pair(s) hypothesized as enriched and an estimate of the (µ, σ) associated with the anticipated signal. Ideally for a BWAS, we would not need to hypothesize which network-pair(s) will be enriched in advance; however, this method is currently limited to estimating power within a single network-pair. Future directions regarding brain-wide power are discussed below.

This work highlighted a limitation of ORA enrichment analysis: its unsuitability for smaller network-pairs. 21 % of the network-pairs had to be excluded for this reason. Expanding and comparing other methods of enrichment analysis may provide an avenue for detecting signals in these smaller network-pairs. Of particular interest for future work are FCS methods, which provide a more continuous measure of enrichment than ORA (Efron and Tibshirani, 2007, Barry et al., 2005), and methods using the competitive null hypothesis, which analyze results across all ROI-pairs jointly (Tian et al., 2005). Another current methodological limitation of ORA enrichment analysis is the arbitrariness of the ORA screening statistic threshold (α). We broadly found that a higher threshold (α=0.95) attained higher power, although a lower threshold (α=0.50) may perform better if there are more constituent ROI-pairs with smaller individual effect. For a prospective analyst, α=0.95 is suggested to begin with, although it may be worthwhile to experiment with alternative values (noting that the Bonferroni corrected significance level should be adjusted accordingly).

The power analyses described here are also limited to power at the network-pair level, since self-contained null hypothesis testing is done independently for each network-pair. Extrapolating to experiment-wide power across all network-pairs in an atlas will be important for prospective power analyses. In future work, we will explore various methods of extrapolating from network-pair power to brain-wide power, from weighted averages to summary statistics of the distribution of powers over network-pairs.

We are currently working on developing an accurate functional model for power estimation to allow for easier execution of power analyses. This, together with extrapolating to brain-wide power, would allow for a more streamlined process for advising on prospective study sample size selection.

As a general guideline for the prospective analyst, statistical power for enrichment analysis is higher for networks with more constituent ROI-pairs. Additionally, smaller networks will likely lead to more network-pairs unsuitable for ORA enrichment analysis. A larger number of total networks will correspondingly increase the number of network-pairs, leading to stricter significance levels when correcting for multiple testing and driving down power. As such, atlases with more ROIs and fewer functional networks are likely to be more suitable to enrichment analysis than smaller atlases with more functional networks.

We also note than any specific numerical results presented in this paper are specific to the data set and atlas (ROI and functional network assignments) used, and power estimates may vary for different ages, populations, ROI maps, or network atlases. Broader trends noted in this paper are expected to be generalizable, such as enrichment analysis outperforming mass univariate analysis and permutation testing being a robust method of significance assessment. It is encouraging that these results parallel those found by Noble et al. when benchmarking a different network-level inference method; in the parlance of this paper, their method is an enrichment analysis with an FCS enrichment statistic (Noble et al., 2022). However, due to the relatively narrow scope of the present paper, we cannot make broad, sweeping claims. While these results are an important and necessary first step, generalization of these findings will come from the study of results from multiple applications to future and different data sets. Repeating these analyses on different data sets, age groups, and atlases remain as a future direction for this work.

Enrichment analysis presents one possible solution to the systemic challenge with reproducibility in fcMRI studies of cross-group differences or brain-behavior associations. This particular enrichment analysis and software implementation allows for a wider range of models and significantly improves the runtime. The statistical results presented here should make researchers feel confident in using this method, providing reassurance in the reported significance values, demonstrating the improved power as compared to mass univariate analysis, and providing an approach to estimate statistical power.

## Funding statement

Funding included R01 MH118362-02S1 (IBIS), R01 HD055741 (IBIS), T32 HD040127 (Ferguson), R01 MH121462 (Pruett), P50 HD103525 (Pruett), R01 MH116961 (Pruett), R01 MH129426 (Pruett), C-BRiMD (Pruett), Eagles Autism Challenge (Pruett), The Drs. John R. (Sr.) and Patricia O. Pruett Fund for Research in Social Cognition and for Undergraduate Training (Pruett), K01-MH122779 (Girault), P50HD103573 (Styner)

## Patient consent and ethics approval statement

Informed written assent was obtained for each subject, and consent and parental permission was obtained from at least one parent of all participants. Participant privacy rights have been properly observed. All study protocols were approved by the University of North Carolina at Chapel Hill’s Institutional Review Board as the lead sIRB site for the IBIS Network (IRB #17–1871, Brain and Behavior Study of Autism from Infancy through School Age, approved 8/21/17)

## CRediT authorship contribution statement

**Pandey Juhi:** Writing – review & editing, Funding acquisition, Data curation. **Girault Jessica:** Writing – review & editing, Methodology, Data curation. **St. John Tanya:** Writing – review & editing, Funding acquisition, Data curation. **Hazlett Heather:** Writing – review & editing, Funding acquisition, Data curation. **Piven Joseph:** Writing – review & editing, Supervision, Project administration, Methodology, Investigation, Funding acquisition, Data curation, Conceptualization. **Schultz Robert:** Writing – review & editing, Funding acquisition, Data curation. **Marrus Natasha:** Writing – review & editing, Funding acquisition, Data curation. **Pruett John:** Writing – review & editing, Visualization, Supervision, Resources, Project administration, Methodology, Investigation, Funding acquisition, Data curation, Conceptualization. **Todorov Alexandre:** Writing – review & editing, Visualization, Validation, Supervision, Software, Methodology, Investigation, Funding acquisition, Formal analysis, Data curation, Conceptualization. **Styner Martin:** Writing – review & editing, Funding acquisition, Data curation. **Torres-Gomez Santiago:** Writing – review & editing, Project administration, Data curation. **Gerig Guido:** Writing – review & editing, Funding acquisition, Data curation. **Evans Alan:** Writing – review & editing, Funding acquisition, Data curation. **Dager Stephen:** Writing – review & editing, Funding acquisition, Data curation. **Estes Annette:** Writing – review & editing, Funding acquisition, Data curation. **Ferguson Austin:** Writing – review & editing, Writing – original draft, Visualization, Validation, Methodology, Formal analysis, Conceptualization. **Zwaigenbaum Lonnie:** Writing – review & editing, Funding acquisition, Data curation. **Nishino Tomoyuki:** Writing – review & editing, Writing – original draft, Visualization, Data curation, Conceptualization.

## Declaration of Competing Interest

Dr. Alan Evans serves as the CSO of Lasso Informatics, which offers databasing and analytics services similar to the LORIS database that underpins IBIS. The other authors report no biomedical financial interests or potential competing interests.

## Data Availability

Data will be made available on request.
